# The Global Prevalence of Infections in Urology Study: A Long-Term, Worldwide Surveillance Study on Urological Infections

**DOI:** 10.3390/pathogens5010010

**Published:** 2016-01-19

**Authors:** Florian Wagenlehner, Zafer Tandogdu, Riccardo Bartoletti, Tommaso Cai, Mete Cek, Ekaterina Kulchavenya, Béla Köves, Kurt Naber, Tamara Perepanova, Peter Tenke, Björn Wullt, Florian Bogenhard, Truls Erik Bjerklund Johansen

**Affiliations:** 1 Department of Urology, Paediatric Urology and Andrology, Justus-Liebig-University, D-35392 Giessen, Germany; 2 Northern Institute for Cancer Research, Newcastle University, Newcastle Upon Tyne NE2 4HH, UK; drzafer@gmail.com; 3 Department of Experimental and Clinical medicine, University of Florence, 4-50121 Florence, Italy; riccardo.bartoletti@unifi.it; 4 Department of Urology, Santa Chiara Regional Hospital, 38122 Trento, Italy; ktommy@libero.it; 5 Department of Urology, Trakya Medical School, Edirne 22100, Turkey; cekmd@doruk.net.tr; 6 TB Research Institute, Novosibirsk 630040, Russia; ku_ekaterina@mail.ru; 7 Jahn Ferenc South Pest Teaching Hospital, 1204 Budapest, Hungary; urologia@jahndelpest.hu (B.K.); tenke.peter@jahndelpest.hu (P.T.); 8 Department of Urology, Technical University of Munich, 80333 Munich, Germany; kurt.naber@nabers.de; 9 S.R. Urology Institute, Moscow 105425, Russia; perepanova2003@mail.ru; 10 Department of Microbiology, Immunology and Glycobiology, Lund University, 22100 Lund, Sweden; bjorn.wullt@med.lu.se; 11 Department of Bioinformatics, Technische Hochschule Mittelhessen, 35390 Giessen, Germany; florian.bogenhard@mni.thm.de; 12 Department of Urology, Oslo University, 0586 Oslo, Norway; t.e.b.johansen@medisin.uio.no

**Keywords:** healthcare-associated urinary tract infections, surveillance study, antibiotic resistance, antibiotic administration, urosepsis, prostate biopsy, transurethral resection

## Abstract

The Global Prevalence of Infections in Urology (GPIU) study is a worldwide-performed point prevalence study intended to create surveillance data on antibiotic resistance, type of urogenital infections, risk factors and data on antibiotic consumption, specifically in patients at urological departments with healthcare-associated urogenital infections (HAUTI). Investigators registered data through a web-based application (http://gpiu.esiu.org/). Data collection includes the practice and characteristics of the hospital and urology ward. On a certain day in November, each year, all urological patients present in the urological department at 8:00 a.m. are screened for HAUTI encompassing their full hospital course from admission to discharge. Apart from the GPIU main study, several side studies are taking place, dealing with transurethral resection of the prostate, prostate biopsy, as well as urosepsis. The GPIU study has been annually performed since 2003. Eight-hundred fifty-six urology units from 70 countries have participated so far, including 27,542 patients. A proxy for antibiotic consumption is reflected by the application rates used for antibiotic prophylaxis for urological interventions. Resistance rates of most uropathogens against antibiotics were high, especially with a note of multidrug resistance. The severity of HAUTI is also increasing, 25% being urosepsis in recent years.

## 1. Introduction

Healthcare-associated infections (HAI) impose a serious threat on the healthcare of patients in terms of morbidity, as well as mortality. The continued increase of the antimicrobial resistance of pathogens worldwide is also a cause of concern, since pathogens do not respect geographical borders. The prevalence and outcome of HAI is an important quality parameter that is routinely collected by monitors of healthcare in a number of countries. Reducing the risk and, hence, prevalence of healthcare-associated infections is a key priority in all healthcare systems, and for this purpose, local and international monitoring is very useful.

**Figure 1:**
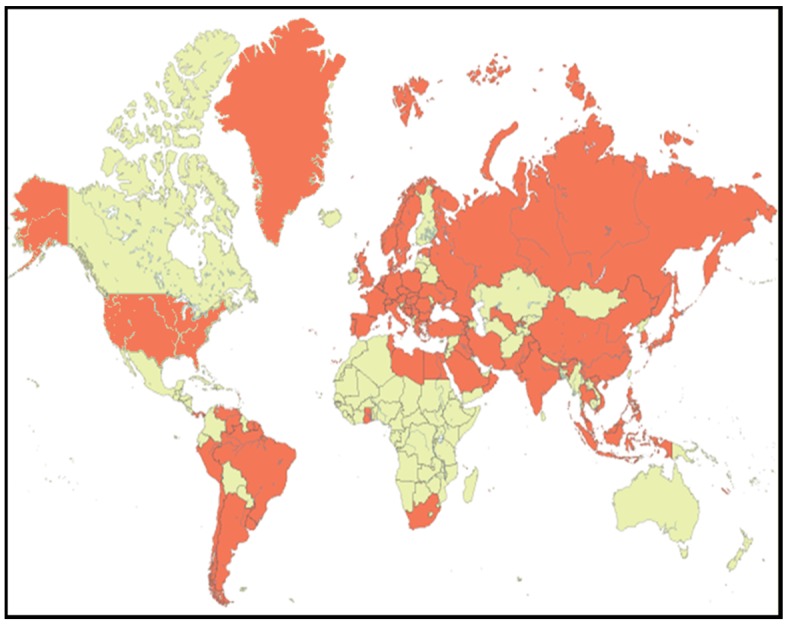
GPIU world map. Participating countries are marked in red.

Healthcare-associated urogenital tract infections (HAUTI) are some of the most-frequently occurring HAI. In a recent U.S.-wide multistate point-prevalence survey, 12.9% of all HAI were due to HAUTI [1]. In a European point prevalence survey conducted by the European Center for Disease Prevention and Control (ECDC), HAUTI accounted for 19.0% of all HAI [2]. These figures, however, may vary significantly in different clinical cohorts. Especially, clinical cohorts with interventions in the urogenital tract are more prone to acquire HAUTI, such as urology. It is therefore important that specific surveillance data are generated for urological patients. Specific data on HAUTI in urology patients, however, are rare. It was therefore that a prevalence study on infections in urological patients was started in 2003 with the aim to deliver surveillance data at the European level first and was called the Pan European Prevalence (PEP) study. In 2004, the study was enlarged to Asia and called the Pan EuroAsian Prevalence (PEAP) study. From 2005 on, the study was run annually and world-wide and was named the Global Prevalence of Infections in Urology (GPIU) study [3] (Figure 1).

The primary aims of the study are to do the following in urology departments throughout the world:

(1) Evaluate urology practice in terms of hospital infection control, which includes:
  - Control programs for catheters, antibiotics, *etc.*;
  - Antibiotic consumption practice.
(2) Evaluate UTI and surgical site infections (SSI) in hospitalised urological patients, which includes:
  - Patient baseline characteristics;
  - Pathogens and their antimicrobial resistance;
  - Antimicrobial treatment.
(3) Determine the prevalence of HAI for:
  - Geographical regions;
  - Varying hospital setting;
  - Study years.

Through these aims, the results of the study will provide national and international data on UTI and SSI for use in further research and will allow individual institutions to bench-mark their performance against national and international peers.

The secondary aims of the study are to offer participating urology departments and urologists:

(1) an instrument for quality control of healthcare-associated infections within their institution;
(2) acknowledgement of active involvement in an infection control program (European Section for Infections in Urology (ESIU)/European Association of Urology (EAU) Certificate for infection control).

## 2. Results and Discussion

The initial findings of the PEP and PEAP studies in 2003 and 2004 showed that the prevalence of HAUTI was 11% in the combined analysis of both initial years [4]. The most frequent forms of HAUTI were asymptomatic bacteriuria in 29%, followed by cystitis in 26%, pyelonephritis in 21% and urosepsis in 12% [4]. Especially, the frequency of urosepsis, however, increased significantly over the last years [5]. This is a worrying figure, also with regard to possible increased mortality, although the contributing factors are not entirely clear, but might include increased age, higher comorbidities and more complicated interventions in this patient population. This finding calls for a high level of awareness in the urological patient population, to note that HAUTI are severe infections, merging into urosepsis in up to 25% [5].

A total of 56% of the hospitalized urological patients were receiving antimicrobial therapy on that study day, of whom 46% received antibiotics for prophylaxis, 26% for microbiologically-proven UTI, 21% for only clinically-suspected UTI and 7% for other infections [6]. The most commonly-used antibiotics were broad spectrum agents, such as fluoroquinolones in 35%, cephalosporins in 27% and penicillins in 16%. Differences between countries and regions, however, were highly significant at that stage [6]. In the follow up studies, a total of 27,542 patients were included in the study on a worldwide global level until now. Routine antibiotic prophylaxis of all urological procedures was highest in Asia, Africa and Latin America with 86%, 85% and 84%, followed by Europe with 67%. Antibiotic prophylaxis was not always consistent with recommended guidelines [7].

Resistance rates of all antibiotics tested other than carbapenems against the total bacterial spectrum were higher than 10% in all regions. The resistance rates of most of the uropathogens against the antibiotics tested did not show significant trends of increase or decrease, but were high already in the beginning years. Resistance to almost all pathogens was lowest in North Europe and highest in Asia [8].

The studies showed that there was a correlation between increased antibiotic use, often with broad-spectrum antimicrobials and increased antimicrobial resistance [9]. This finding is in line with the observations that increased antibiotic consumption, especially of antibiotic agents with the propensity for collateral damage on the microbiome, leads to antibiotic resistance and multi-resistance. To interrupt such a vicious cycle, our results suggested that there is room for improvement in surgical prophylaxis in terms of limiting exposure to antibiotics and that too many patients with asymptomatic bacteriuria were treated with antibiotics [9]. In addition, a bundle of tools will need to be implemented to slow down the emergence of resistance, such as antimicrobial stewardship and implementation of non-antibiotics strategies in benign infections, amongst others.

Emerging data showed that infection is a serious adverse effect of prostate biopsy; therefore, we performed a prostate biopsy side study with the aim to prospectively evaluate the incidence of infective complications after prostate biopsy and identify risk factors in the years 2010 and 2011 [10]. In a total of 702 men included from 84 GPIU participating centres worldwide with outcome data available for 521 men, symptomatic UTI was seen in 5%, febrile UTI in 3.5%, and 3% required hospitalisation. Multivariate analysis did not identify any patient subgroups at a significantly higher risk of infection after prostate biopsy. This side study also confirmed a high incidence of fluoroquinolone resistance in causative bacteria [10].

The detailed results of the side study on TURP will be presented elsewhere.

Given the fact that HAUTI are a significant clinical problem, especially in urology, the rising antimicrobial resistance calls for a closer monitoring of HAUTI on an international level within urology [11]. In order to meet these challenges, the ESIU has been performing this prevalence study with great success for more than 11 years now. It is also a quality improvement initiative related to HAUTI. The study is able to demonstrate what risk factors are important and how these risk factors are developing over time. Although the study is a prevalence study, it incorporates some longitudinal aspects, as patients are evaluated throughout their full hospital course, which resembles a unique study design, creating valuable data.

The long-term course of the study since 2003 also enabled detecting important emerging issues, such as infectious complications after prostate biopsy or the fact that the severity of HAUTI has increased. This led to the development of side studies that explicitly deal with these new emerging problems, in order to prospectively create data, possibly leading to changes in clinical practice.

## 3. Experimental Section

### 3.1. Study Design

The GPIU study is a multinational, multicentre study, performed annually since 2003 as a one-day prevalence study in November of each year. This time of the year was chosen to have a uniform time period across the world. The study was initiated and organized by the board of the European Section for Infections in Urology (ESIU), a section of the European Association of Urology (EAU). The study was endorsed by the ESIU and sponsored by the EAU. The study is a web-based application, and data are delivered online. Participating centres provide information regarding their hospital and urology ward characteristics and practice.

### 3.2. Study Day Allocation

Each participating department can freely choose a single study day within several listed periods during November and December of each year. On the chosen single study day at 08:00 a.m. local time, all patients present in the ward should be screened and included. The presence of UTI and/or SSI according to the Centers for Disease Control (CDC) definitions during their entire hospital stay should be documented and audited, encompassing the patients’ full hospital course from admission to discharge. The investigators have to state for each reported patient whether the infections is a HAUTI or not. Thus, the charts and case records of the included patients should be examined both retrospectively and prospectively and patients categorized as having or not having a UTI or SSI. All uploaded patient information is reported anonymously to the central study file. All participating departments are allocated their own study page where their patients are listed anonymously according to subject numbers. Data of the years 2003 and 2004 were combined into one group due to data file structure changes made in 2005.

### 3.3. Internet Application

Study-report forms are available through the GPIU portal at http://gpiu.esiu.org/ [12].

**Figure 2:**
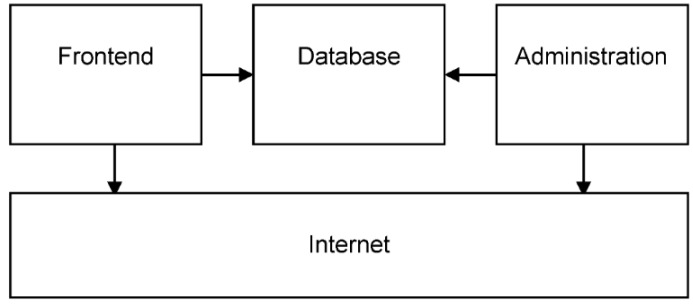
Structure of the Global Prevalence of Infections in Urology (GPIU) application on the Internet.

An original Internet application has been developed and programmed in PHP (a recursive acronym for PHP Hypertext Preprocessor). The structure of the application is shown in Figure 2. Investigators fill in reply forms on a separate page (the so-called frontend). Inputted data are stored securely in a specially-designed MySQL database. Care has been taken to separate individual investigator data strictly and to guarantee optimal privacy. The structure of the GPIU study application is designed to be adapted to other Internet-based clinical studies in the future.

**Figure 3:**
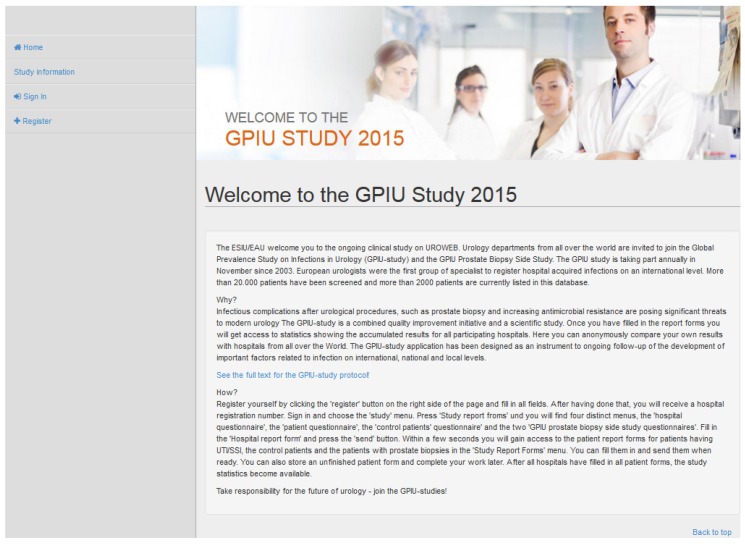
Welcome page of the GPIU study.

The first page of the website (welcoming page) presents basic information about the GPIU study and its aims (Figure 3). An investigator registers on this page. Subsequent to registration, the investigator will gain access to the web portal of the study with a username and personal password. Each investigator/department is allocated a centre number and patient-unique study numbers through the study website. The study web portal allows the investigator to navigate to data entry and help functions from a single page, and he or she can fill in study forms, consult help pages and alter his or her personal information.

The predefined workflow of the study is reflected on to the web-portal. The steps that are linked with each other and occur in tandem as follows: (i) registration to the study; (ii) input of hospital and department information; and (iii) input of patient forms with infection (case report forms). Investigators can fill in the patient forms, save the information and continue entries at another time. A button for “validate” will appear only after all questions in the patient form are filled. Subsequent to the user actively clicking on the “validate” button, the data will be submitted to the main patient registry file.

### 3.4. Study Variables

Each participating department first completes the “Hospital Registration Form”, which details the population of patients hospitalized in that department at 8:00 a.m. on the chosen study day. Departments who previously participated will be provided with the “Hospital Registration Form” of the previous year and are asked to update the information for each study year. An individual “Patient Registration Form” is then completed for each patient categorized as having UTI or SSI (according to CDC criteria [13]) and hospitalized in the participating urology department at 8:00 a.m. on the chosen study day. Definitions of all requested variables included on the different forms are available by means of help buttons.

Variable groups that are collected within the hospital and department forms are as follows:

- Geographical location
- Hospital size, setting, case volume
- Hospital and department infection control program
- Study day patient numbers (hospital and departmental)
- Urology department antibiotic practice program
- Study day antibiotic consumption

Variable groups that are collected within the patient forms are as follows:

- Demographics
- Comorbidities using the Charlson comorbidity score
- Interventions performed in the patient
- Antibiotics used
- Urine/surgical site/blood culture results
- Antimicrobial treatment for a current episode of infection (if given).

### 3.5. GPIU Side Studies

Apart from the GPIU main study, several side studies are/were taking place, dealing with transurethral resection of the prostate (TURP), prostate biopsy, as well as urosepsis.

#### 3.5.1. Prostate Biopsy Side Study

Since 2010, a side study designed to audit the prevalence of infective complications following prostate biopsy has been carried out. Prostate biopsy is an extremely valuable and frequently performed diagnostic procedure in urology. There is some evidence that infective complications following prostate biopsy are increasing in number and severity in many countries possibly related to increased resistance of faecal pathogens to antibiotics, such as fluoroquinolones used for prophylaxis. The GPIU prostate biopsy side study is a prevalence study on infective complications of prostate biopsy to audit the prevalence of infective complications after prostate biopsy across centres and countries participating in the GPIU main study. Risk factors associated with a higher risk of infective complications are evaluated, and changing resistance patterns to antibiotics used for prophylaxis are determined. Furthermore, the prostate biopsy side study evaluates the antimicrobial management of patients with post-biopsy infection and their clinical outcomes.

##### Prostate Biopsy Study Design

All patients undergoing prostate biopsy during the 2-week period commencing on the GPIU study day chosen by each centre should be included and followed up. Subsequent to registering patients to the study, they are contacted 14 days following their biopsy and interviewed regarding infective complications, either by telephone or face-to-face. The investigator for each centre will complete a participant data file for each patient, including detailing relevant pre-biopsy characteristics, the biopsy protocol followed and infective complications during the 14-day period following biopsy.

#### 3.5.2. Transurethral Resection of the Prostate Side Study

From 2006 to 2009, a side study designed to audit the prevalence of infective complications following transurethral resection of the prostate (TURP) was performed. The aim of this study was to determine the prevalence of HAUTI and other complications of patients in the GPIU study that received a TURP. Interventional and patients’ specific risk factors were evaluated.

#### 3.5.3. Urosepsis Side Study (Serpens Study)

Comparing the different HAUTIs between 2003/2004 and 2008 showed that severe infections, such as pyelonephritis and urosepsis, were more prevalent in 2008 compared to 2003/2004. A further investigation of the subcohort of patients with urosepsis from 2003 to 2013 corroborated this finding and led to the design of a prospective study on urological patients with urosepsis, which is currently being conducted.

### 3.6. Microbiological Investigations

All urine cultures and other microbiological investigations are conducted in the local laboratories according to their microbiological standards. Information about the standard used for antimicrobial susceptibility testing is provided by most of the centres.

### 3.7. Statistical Analysis of Data

Study data were imported from the web-based survey into Microsoft Access (Microsoft Corp, Seattle, WA, USA), as a comma separated file (csf), and reorganized. The data files were transferred to statistical packages for analysis.

The initial review of each year’s data is carried out descriptively. Followed by this, pooled analysis of data is carried out in a similar manner to identify any emerging trends. Finally, the pooled data are further carried into statistical modelling to explain any trends that may have been identified.

In the case of antimicrobial susceptibility changes over the years, resistance rates are specified by bacterial species and antibiotics tested for each consecutive year. Geographic differences were assessed according to 4 regions (North Europe, South Europe, Asia, Africa + South America (for statistical reasons due to low case numbers, Africa and America were merged)). South European countries were defined as European countries with a shore on the Mediterranean Sea and North Europe as the remaining European countries.

## 4. Conclusions

The GPIU study is an annual, worldwide-conducted prevalence study to survey infections in urological patients on an annual basis since 2003. Antibiotic resistance rates are very high in all locations, and antibiotic usage is not always optimal. Therefore, this study can help to deliver data for guideline recommendations of adequate empirical antibiotic therapy in hospitalized urological patients. Knowledge of regional and local resistance data and prudent use of antibiotics, however, continue to be important strategies to optimize antibiotic therapy in urological patients with infections.
